# Dietary saturated fat/cholesterol, but not unsaturated fat or starch, induces C-reactive protein associated early atherosclerosis and ectopic fat deposition in diabetic pigs

**DOI:** 10.1186/1475-2840-10-64

**Published:** 2011-07-14

**Authors:** Sietse J Koopmans, Ruud Dekker, Mariette T Ackermans, Hans P Sauerwein, Mireille J Serlie, Heleen MM van Beusekom, Mieke van den Heuvel, Wim J van der Giessen

**Affiliations:** 1 BioMedical Research of Wageningen University and Research Center, Lelystad, The Netherlands; 2 Department of Animal Sciences, Adaptation Physiology Group of Wageningen University, Wageningen, The Netherlands; 3 Clinical Chemistry, Laboratory of Endocrinology, Academic Medical Center, Amsterdam, The Netherlands; 4 Endocrinology & Metabolism, Academic Medical Center, Amsterdam, The Netherlands; 5 Experimental Cardiology, Erasmus Medical Center, Rotterdam, The Netherlands; 6 ICIN-KNAW, Utrecht, The Netherlands

**Keywords:** Diabetes, Insulin, Diet, Unsaturated fat, Saturated fat, Cholesterol, Inflammation, C-reactive protein, Atherosclerosis, Pigs

## Abstract

**Background:**

Diabetes is thought to accelerate cardiovascular disease depending on the type of diet. This study in diabetic subjects was performed to investigate the metabolic, inflammatory and cardiovascular effects of nutritional components typically present in a Western, Mediterranean or high glycaemic diet.

**Methods:**

Streptozotocin-diabetic pigs (~45 kg) were fed for 10 weeks supplemental (40% of dietary energy) saturated fat/cholesterol (SFC), unsaturated fat (UF) or starch (S) in an eucaloric dietary intervention study.

**Results:**

Fasting plasma total, LDL and HDL cholesterol concentrations were 3-5 fold higher (p < 0.01) in SFC compared to UF and S pigs. Fasting plasma NEFA concentrations (mmol/L) were highest (p < 0.05) in SFC (1.09 ± 0.17), intermediate in UF (0.80 ± 0.14) and lowest in S pigs (0.58 ± 0.14) whereas plasma glucose (~13 mmol/L), triglyceride (~0.5 mmol/L) and insulin (~24 pmol/L) concentrations were comparable among SFC, UF and S pigs. The postprandial response area under the curves (AUC, 0-4 h) for glucose but not for insulin and triglyceride responses were intermediate in SFC (617 ± 144) and lowest (p < 0.05) in UF (378 ± 157) compared to S pigs (925 ± 139). Fasting hepatic glucose production, hepatic and peripheral insulin sensitivity and blood pressure were not different among pigs. C-reactive protein (CRP) concentrations (mg/L) were highest (p < 0.05) in SFC (25 ± 4), intermediate in S (21 ± 3) and lowest in UF pigs (14 ± 2). Liver weights, liver and muscle triglyceride concentrations, and the surface area of aorta fatty streaks were highest (p < 0.01) in SFC pigs. A positive correlation between postprandial plasma CRP and aorta fatty streaks was observed in SFC pigs (R^2 ^= 0.95). Retroperitoneal fat depot weight (g) was intermediate in SFC (260 ± 72), lowest in S (135 ± 51) and highest (p < 0.05) in UF (571 ± 95) pigs.

**Conclusion:**

Dietary saturated fat/cholesterol induces inflammation, atherosclerosis and ectopic fat deposition whereas an equally high dietary unsaturated fat load does not induce these abnormalities and shows beneficial effects on postprandial glycaemia in diabetic pigs.

## Background

The impact of an excess in dietary fats and carbohydrates on metabolic control, inflammation and cardiovascular disease has been studied and discussed in normal and (pre)diabetic subjects in both human [1-4] and animal studies [5-7]. In general, excessive dietary saturated fats and cholesterol increase the risk for the development of obesity, diabetes and cardiovascular diseases [6,8-10] while dietary unsaturated fats are considered less harmful and do not impose an increased risk for the development of diabetes and cardiovascular diseases [1,2,6]. Dietary carbohydrates, in the form of starches, have a high glycaemic load and thereby worsen postprandial glucose, stimulate insulin secretion and de novo lipogenesis [11-14].

Most studies on the longer term (months) effects of dietary components have been carried out in normal or glucose intolerant individuals but limited information is available in diabetic subjects on metabolic control, inflammation, cardiovascular abnormalities and body composition [2,12,13,15].

Longer term studies in diabetic humans are difficult to perform because adherence to the prescribed diets has proven to be extremely difficult [16] and because dietary effects on the pathophysiology of diabetes are usually small with respect to metabolic control, insulin sensitivity, inflammation and cardiovascular diseases [17,18]. In addition, longer term studies are usually necessary to disclose any dietary effects on the pathophysiology of diabetes because part of the dietary effects are caused by changes in body composition. This can partly be overcome by studying animal models which are representative for the human situation, are highly homogenous and are kept under strictly standardized experimental conditions.

We have developed a pig model for diabetes mellitus type 2 in humans which is characterized by insulin resistance, hyperglycaemia as well as elevated plasma triglyceride and NEFA concentrations. The diabetic pigs are non-ketotic, anabolic and do not require insulin therapy [19]. Pigs are like humans omnivores and as such, the functionality of the gastrointestinal tract is comparable between pigs and man and therefore the pig is an useful animal model for the study of dietary components [20,21]. This makes the pig particular useful when the effect of diets is studied on diabetes accelerated dyslipidaemia and atherosclerosis [22-24]. Furthermore, coronary arteries of diabetic pigs have been shown to express low grade inflammation [25], a condition also described in humans with type 2 diabetes mellitus [13,26,27].

The aim of this dietary intervention study was to characterize and compare the medium/long-term (10 weeks) pathogenic effects of eucaloric diets 1) both high in supplemental fat, but differing in fat composition as reflected by mainly saturated fats and cholesterol (SFC) versus mainly unsaturated fats (UF) and 2) differing in supplemental fat (SFC or UF) versus supplemental carbohydrate (starch, S), on pre- and postprandial hyperglycaemia, lipidaemia and insulinaemia, on insulin sensitivity, blood pressure, circulating pro-inflammatory markers, retroperitoneal fat weight and on muscle, liver and aorta lipid deposits in diabetic pigs.

## Methods

The performed research is in compliance with the ARRIVE guidelines on animal research [28]. Experimental protocols describing the management, surgical procedures, and animal care were reviewed and approved by the ASG-Lelystad Animal Care and Use Committee (Lelystad, The Netherlands).

### Animals, housing, diets and surgery

Domestic (Landrace × Yorkshire, D-line) pigs (barrows with an initial age and body weight of ~11 weeks and ~30 kg, respectively) were obtained (Bastiaanse, Espel, The Netherlands) and kept in specially designed metabolic pens (1.15 × 1.35 m) and adapted to the light/dark cycle (lights on at 05:00 h and off at 19:00 h) and a feeding schedule. Pigs were weighed weekly and the meal size was adjusted to the individual pig's weight. The pigs were fed 2.5-fold maintenance requirements for gross energy (GE) as established in a normal pig. This corresponded with a feeding level of 1045 kJ GE kg^-1 ^BW^0.75 ^(metabolic weight of the pigs) per day and is sufficient to ensure moderate growth in normal pigs [29].

The pigs were fed a commercial pig diet (5% crude fat, 16% crude protein, 41% starch and sugars, 20% non-starch polysaccharides, 6% ash and 12% water; Startbrok; Agrifirm, Meppel, The Netherlands) twice daily (at 06:00 and 16:00 h). Water was always available ad libitum. After 1 week, pigs were provided with a permanent blood vessel catheter in the jugular vein, as previously described by us [29,30]. One week after surgery, 21 pigs were treated with streptozotocin (140 mg/kg) as described previously [19]. Two weeks thereafter, 3 pigs showed fasting plasma glucose concentrations <10 mmol/L and were excluded from the study. The remaining 18 pigs were balanced over the 3 diet groups, based on fasting hyperglycaemia. Composition of the saturated fat/cholesterol, unsaturated fat and starch enriched diets is shown in Table 1. The experimental meals were fed twice daily (at 1045 kJ GE kg^-1 ^BW^0.75 ^per day) for a duration of 10 weeks. In practical terms this means that the pigs on the starch-enriched diet were fed 1.31-fold the amount (on weight basis, but equal in caloric content) compared to the pigs on the fat-enriched diets in order to match the energy intake of pigs among diets.

**Table 1:** Composition of the experimental diets

Food ingredients	Saturated fat diet plus cholesterol	Unsaturated fat diet	Starch diet
**Milk protein (caseinate)**	23.2	23.2	17.7
**Fructose**	9.2	9.2	7.1
**Maltodextrin DE 19**	30.8	30.8	23.6
**Native pea starch**	0.0	0.0	41.4
**Total sugar and starch**	40.0	40.0	72.1
**Soy bean oil**	0.0	0.0	2.7
**Lecithin**	0.0	1.5	0.0
**Canola oil (rape oil/Lear)**	0.0	2.7	0.0
**Trisun 80, high oleic sunflower oil**	0.0	23.0	0.0
**Beef tallow**	27.2	0.0	0.0
**Total fat**	27.2	27.2	2.7
**Fiber: Fibrim 2000**	9.4	9.4	7.3
**Premix^1^**	0.20	0.20	0.15
**Cholesterol^2^**	1.0	0.0	0.0

**Total (%)**	100.0	100.0	100.0

**Gross Energy (GE; MJ/kg)**	23.4	23.4	17.9

The per-cent of each ingredient contributing to the total amount of diet (= 100%) is listed. Energy intake was matched among dietary groups and therefore pigs receiving the Starch diet were fed 1.32 times the amount of the high fat diet groups. Therefore the diets were homogenous except for starch or fat.

^1 ^This vitamin and trace mineral premix contained per kg diet: vit. A (retinol) - 1750 IU; vit. D_3 _(cholecalciferol) - 200 IU; vit. E (tocopherol) - 11 IU; vit. K_1 _(phylloquinone) - 0.5 mg; vit. B_1 _(thiamin) - 1.0 mg; vit. B_2 _(riboflavin) - 4 mg; d-pantothenic acid - 9 mg; niacin (vit. B_3_, nicotinic acid) - 12.5 mg (available); biotin (vit. H) - 50 μg; vit. B_12 _(cyanocobalamin) - 15 μg; folic acid (folacin) - 0.3 mg; vit. B_6 _(pyridoxin) - 1.5 mg; choline - 400 mg; Fe - 80 mg; Zn - 54 mg; Mn - 30 mg; Co - 0.15 mg; I - 0.14 mg; Se - 0.25 mg; antioxidants (E310,320,321) - 50 mg; with maize starch as carrier.

^2 ^Cholesterol has no energetic value and therefore 1 kg cholesterol was supplemented per 100 kg diet.

Supplemental saturated fat plus cholesterol (SFC), unsaturated fat (UF), or starch (S) content was 40% of total dietary energy. SFC was derived from beef tallow (i.e. ~55% saturated, ~35% mono-unsaturated, and ~10% poly-unsaturated fatty acids) plus 1% cholesterol, UF was derived from sunflower and rape oil (i.e. ~65% poly-unsaturated, ~25% mono-unsaturated, and 10% saturated fatty acids) and S was derived from pea starch, representing components of a Western, Mediterranean or high glycaemic diet, respectively.

### Timeline

**Figure 1:**
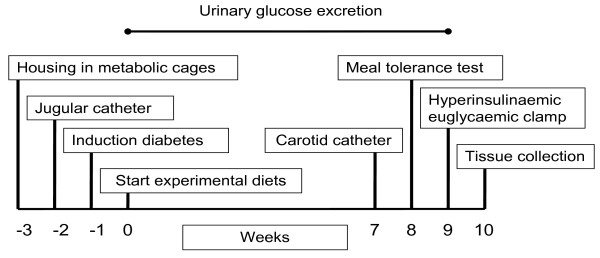
**Timeline of the study**.

The timeline of the study is represented schematically in Figure 1. In short, the 3 diets were fed to the pigs (n = 6 per diet group) for 10 weeks comprising the following techniques and measurements: 1) daily food intake, 2) weekly body weight, 3) quantitative collection of urine (for determination of 24-h urinary glucose excretion) on Mondays, Wednesdays and Fridays during the dietary treatment, 4) at week 7, the pigs were provided with permanent catheters in the jugular vein and carotid artery, 5) at week 8, a meal tolerance test was performed and intra-arterial blood pressure was recorded, 6) at week 9, a hyperinsulinaemic euglycaemic clamp study using 6,6-^2^H_2_-glucose infusion was performed and 7) at week 10, pigs were killed by intravenous injection of barbiturate (administered via the jugular catheter) for tissue collection.

### Meal tolerance test and blood pressure

During the one-week recovery period after surgery, the pigs were habituated to the blood sampling and experimental procedures. The carotid artery catheter was used for blood sampling and registration of blood pressure. During the blood sampling procedure, the catheters were flushed with physiological saline and sealed off with physiological saline containing 5 IU heparin per mL. From experience we know that this sampling procedure does not affect plasma NEFA concentrations.

Blood was sampled repeatedly before, during and after the morning meal (-15, 0, 30, 60, 120, 240, 360, and 480 minutes from the start of feeding). Per sampling time point, approximately 5 ml blood was sampled. The responses of insulin, glucose and triglycerides were assessed. In addition, the pre-and post-prandial levels of C-reactive protein (CRP), haptoglobin, IL-6 and TNF-α were determined on samples taken 15 minutes before and 180 minutes after the start of feeding. Subsequent to the meal tolerance test, the blood pressure and heart rate were determined five hours post-prandially on a Digital Electromanometer, Type 330 (Hugo Sachs Elektronik KG, March-Hugstetten, Germany).

### Hyperinsulinaemic euglycaemic clamp and 6,6-^2^H_2_-glucose infusion

The jugular vein catheter was used for the infusion of fluids and the carotid artery catheter was used for blood sampling during the hyperinsulinaemic clamp study. Preceding the insulin clamp, overnight fasting blood samples were collected for determining the concentrations of fructosamine, glucagon, cortisol, NEFA, total cholesterol, LDL and HDL cholesterol and background glucose enrichment.

After baseline samples, a prime (4.8 mg/kg)-continuous (0.08 mg.kg^-1^.min^-1^) infusion of 6,6-^2^H_2_-glucose was administered for 150 min. After an equilibration time, blood samples were taken at 110, 120, 130, 140 and 150 min for determination of glucose, 6,6-^2^H_2_-glucose enrichment (to estimate fasting hepatic glucose production) and insulin. Subsequently, insulin was infused (prime (34 mU/kg)-continuous (2 mU.kg^-1^.min^-1^)) for 6 hours. A variable infusion of a 33% D-glucose solution was started to maintain plasma glucose at euglycaemia (~5 mmol/L). Steady state calculations were carried out during the last 40 minutes of the clamp (t = 320, 330, 340, 350 and 360 min) and the coefficients of variation for the insulin and glucose concentrations, and for the infusion rate of glucose were determined. During the last 3 hours of the insulin clamp, a prime (4.8 mg/kg)-continuous (0.08 mg.kg^-1^.min^-1^) infusion of 6,6-^2^H_2_-glucose was superimposed to estimate 1) insulin stimulated whole body glucose uptake (rate of disappearance = Rd) and 2) insulin-inhibited hepatic glucose production, as described before [19]. For this purpose blood samples were taken at t 320, 330, 340, 350 and 360 min of the clamp.

Infusates: Insulin (Actrapid MC, porcine monocomponent, Novo, Copenhagen, Denmark), 6,6-^2^H_2_-glucose (Cambridge Isotope Laboratories, Inc, MA, USA) and D-glucose (Merck, Darmstadt, Germany) were prepared as sterile solutions and passed through a 0.22 μm Millipore filter into sterile containers before use. Insulin was diluted in a saline solution containing pig plasma (final plasma concentration was 3%) in order to avoid sticking of insulin to the plastic containers and tubings. 6,6-^2^H_2_-glucose was dissolved in a saline solution and D-glucose was dissolved in aqua dest.

### Plasma, urine and tissue collection and analyses

Blood samples collected in heparinised (150 USP. U. Lithium Heparin, 10 mL Venoject, Terumo, Leuven, Belgium) or EDTA (ethylenediaminetetraacetic acid, (0.47 mol/L EDTA, 10 mL Venoject, Terumo, Leuven, Belgium) tubes were immediately chilled at 0°C on water with ice, and centrifuged at 4°C for 10 minutes at 3000 rpm. Plasma aliquots were stored at -80°C for later analyses. Urine was quantitatively collected per 24 hours in buckets containing 0.5 grams Halamid-d (sodium-p-toluenesulfonchloramide, Akzo Nobel Chemicals, Amersfoort, The Netherlands) to prevent microbial breakdown of glucose. Urine samples were stored at -20°C for later glucose analyses. Muscle (m. iliopsoasis) and liver were snap-frozen in liquid nitrogen and stored at -80°C. Abdominal aorta was fixed in a 4% paraformaldehyde solution.

Plasma samples for determination of 6,6-^2^H_2_-glucose enrichment were analyzed as described previously [19]. In short, glucose was extracted from plasma, derivatised, and injected into a gas chromatograph/mass spectrometer system (HP 6890 series GC system and 5973 Mass Selective Detector, Palo Alto, CA, USA). Separation was achieved on a J&W scientific DB 17 capillary column (30 m × 0.25 mm × 0.25 μm; Agilent Technologies Nederland BV, Amstelveen, The Netherlands). Isotopic enrichment was calculated as tracer-to- tracee ratio after subtracting the isotopic enrichment of a background plasma sample. An aliquot of the 6,6-^2^H_2_-glucose infusate was analyzed for the isotope concentration to calculate the actual infusion rate for each infusion experiment.

Plasma glucose was analyzed with the Glucose liquiUV mono kit (Human, Wiesbaden, Germany), plasma non-esterified fatty acids were analyzed with the WAKO kit (Neuss, Germany) and plasma triglycerides with a kit from Human (Wiesbaden, Germany). Total, LDL and HDL cholesterol concentrations in plasma were determined with liquicolor kits (Human, Wiesbaden, Germany) and VLDL cholesterol was calculated as total cholesterol minus LDL and HDL cholesterol. Plasma insulin concentration was measured using a Delfia assay (test kit by Perkin Elmer Life Sciences Trust by Wallac Oy, Turku, Finland). This specific pig insulin assay was validated using pig insulin standards, as indicated before [30]. Plasma glucagon was measured with a kit from Euro-Diagnostica (Arnhem, The Netherlands), plasma cortisol with the Count-A-Count Cortisol kit (DPC, Los Angeles, USA) and fructosamine by a kit from Spinreact (Sant Esteve De Bas, Spain). Plasma C-reactive protein (CRP), interleukin-6 (IL-6) and tumor necrosis factor alpha (TNF-α) were analyzed with kits (CRP-hs, Human, Wiesbaden, Germany; Haptoglobin, Instruchemie, Delfzijl, The Netherlands; SW Interleukin 6, IBL, Hamburg, Germany and SW TNF-alpha Elisa Kit, Biosource Int, Camarillo, USA), respectively.

Ketones (acetoacetic acid) were determined in fresh urine by a reagent strip test (Ketostix, Bayer Diagnostics, Mijdrecht, The Netherlands).

Triglyceride concentrations in muscle and liver samples were determined with the same kit as used for the plasma samples, after saponification with an alkalin alcohol solution as previously described [31].

Aortic fatty streaks, as a marker of early atherosclerosis [22,24], (AHA class 2 lesion [32]), were quantified with use of Sudan IV fat stain in sections of the abdominal aorta ranging from the bifurcation of the renal arteries to the bifurcation of the iliac arteries. The stained aortas were then photographed and analyzed with a microscopy image analysis system (Clemex technologies Inc., Quebec, Canada) as ratio of stained area to total area.

### Statistical analyses

Results are expressed as means ± SEM and the criterion of statistical significance was set at p < 0.05. The data were subjected to the analysis of variance procedure (ANOVA) (for repeated measurements when applicable) followed by the unpaired or paired student's t-test of Genstat 5 [33] for determination of differences between or within the three dietary groups, respectively.

## Results

### Body composition

Final body weights were comparable among SFC, UF and S pigs indicating that average food intake and urinary glucose excretion were in balance during the course of the study per diet group (Table 2). However, liver weights and triglyceride concentrations were higher (p < 0.01) in SFC pigs compared to UF and S pigs. Triglyceride concentration in muscle was highest (p < 0.01) in SFC pigs, intermediate in UF and lowest in S pigs. Retroperitoneal fat depot weight was intermediate in SFC (260 ± 72 g), lowest in S (135 ± 51 g) and highest (p < 0.05) in UF (571 ± 95) pigs.

**Table 2:** Food intake, urinary glucose excretion, final body weights and tissue data of diabetic pigs fed saturated fat/cholesterol or unsaturated fat or starch enriched diets

	Saturated fat plus cholesterol diet	Unsaturated fat diet	Starch diet
**Factual average food intake (MJ/day)**	23.9 ± 1.3	25.0 ± 2.0	27.7 ± 0.9
**Average urinary glucose excretion (MJ/day)**	8.5 ± 1.0^A^	9.1 ± 1.1^A^	15.8 ± 2.1^B^
**Final body weight (kg)**	41 ± 3	45 ± 5	48 ± 7
**Liver weight (kg)**	1.47 ± 0.08^A^	1.12 ± 0.03^B^	1.11 ± 0.03^B^
**Liver triglyceride concentration (g/kg)**	49 ± 11^A^	27 ± 1^B^	21 ± 3^B^
**Muscle triglyceride concentration (g/kg)**	21 ± 4^A^	13 ± 3^AB^	8 ± 1^B^
**Retroperitoneal fat weight (g)**	260 ± 72^AB^	571 ± 95^A^	135 ± 51^B^
**Surface area aorta fatty steaks (%)**	17.9 ± 4.4^A^	0.03 ± 0.02^B^	0.14 ± 0.07^B^

^A, B ^, data with different superscripts in the same row differ at p < 0.05.

### Metabolic control

**Figure 2:**
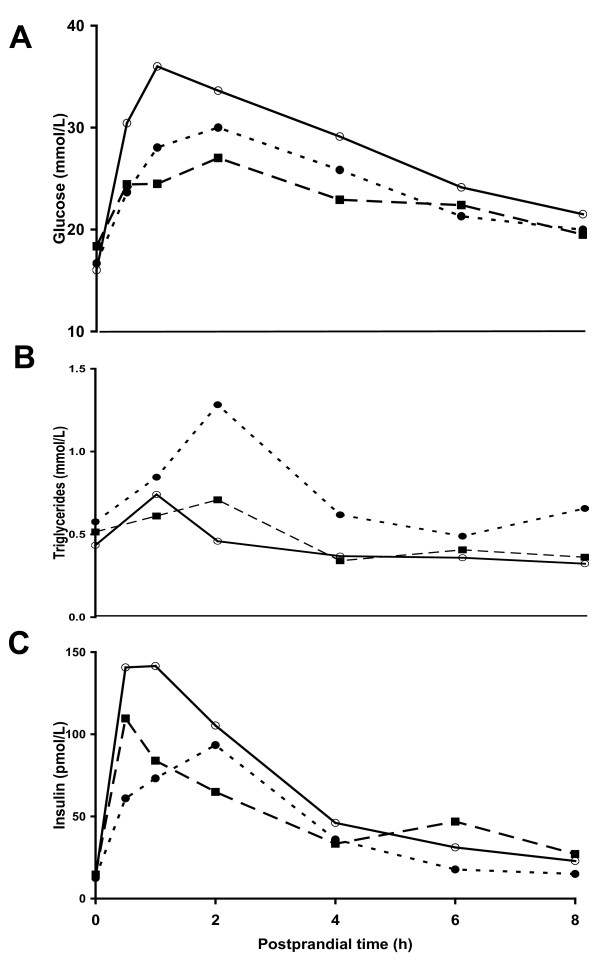
**Postprandial curves (0-8 h) of plasma glucose (A), triglycerides (B) and insulin (C) are shown for diabetic pigs which were fed diets supplemented with starch (open circles), unsaturated fats (black squares) or saturated fats with cholesterol (black circles)**.

**Figure 3:**
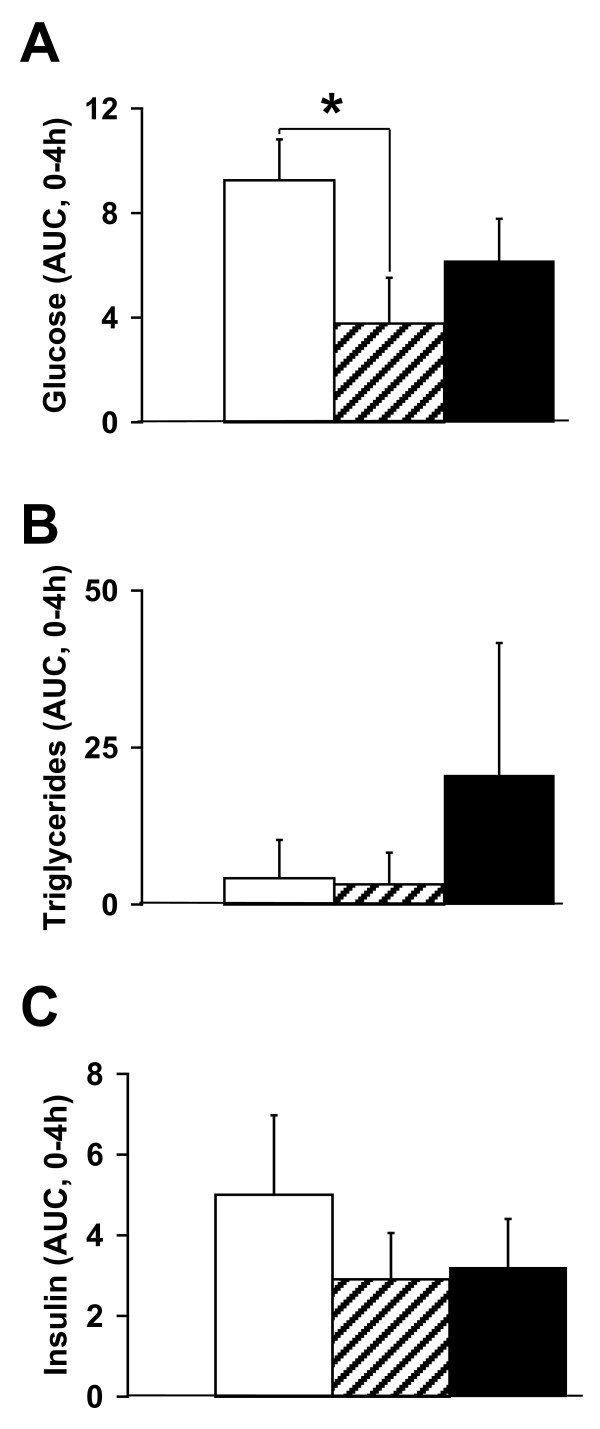
**The postprandial response areas under the curve (Δ0-4 h) are depicted for plasma glucose (A), triglycerides (B) and insulin (C) for supplemental starch (open bars), unsaturated fats (striped bars) or saturated fats with cholesterol (black bars)**. Means ± SEM. *p < 0.05.

At the end of the dietary intervention, fasting plasma insulin, glucagon, glucose, fructosamine and triglyceride concentrations were similar among all diabetic pigs (Tables 3 and 4). Fasting plasma total cholesterol, HDL, LDL and VLDL concentrations were higher (p < 0.01) in SFC pigs compared to UF and S pigs. The ratio of HDL/LDL cholesterol was unchanged but the ratio of HDL/total cholesterol was reduced (p < 0.05) in SFC and UF pigs compared to S pigs (Table 4). Fasting plasma NEFA concentrations were highest (p < 0.05) in SFC pigs, intermediate in UF pigs and lowest S pigs (Table 4). Fasting hepatic glucose production, insulin-inhibited hepatic glucose production and insulin-stimulated whole body glucose uptake were similar among diabetic pigs (Table 3). Coefficients of variation of clamp plasma glucose, plasma insulin and glucose infusion rate in SFC versus UF versus S pigs were 5 ± 2 versus 7 ± 3 versus 3 ± 1%, 11 ± 5 versus 7 ± 2 versus 8 ± 3% and 2 ± 1 versus 3 ± 1 versus 2 ± 1%, respectively. The postprandial response area under the curves (0-4 h or 0-8 h) for glucose but not for insulin and triglyceride responses were intermediate in SFC (617 ± 144 or 471 ± 122), lowest (p < 0.05) in UF (378 ± 157 or 292 ± 133) and highest in S pigs (925 ± 139 or 724 ± 92 (Figures 2 and 3).

**Table 3:** Fasting hepatic glucose production, insulin-stimulated whole body glucose uptake, insulin-inhibited hepatic glucose production and plasma concentrations of metabolites and insulin in diabetic pigs fed saturated fat/cholesterol or unsaturated fat or starch enriched diets

	Saturated fat plus cholesterol diet	Unsaturated fat diet	Starch diet
**Fasting plasma fructosamine (μmol/L)**	449 ± 25	494 ± 62	558 ± 103
**Fasting plasma glucose (mmol/L)**	12.4 ± 0.4	14.4 ± 0.7	12.7 ± 1.6
**Fasting plasma insulin (pmol/L)**	23 ± 7	26 ± 7	22 ± 4
**Fasting hepatic glucose production (mg.kg^-1^.min^-1^)**	7.4 ± 0.8	6.4 ± 0.5	8.2 ± 2.0
**Clamp plasma glucose (steady state, mmol/L)**	5.7 ± 0.2	5.4 ± 0.2	5.4 ± 0.2
**Clamp plasma insulin (steady state, pmol/L)**	320 ± 27	409 ± 33	327 ± 33
**Insulin-stimulated whole body glucose uptake (mg.kg^-1^.min^-1^)**	10.0 ± 2.3	9.5 ± 2.1	10.8 ± 2.8
**Insulin-inhibited hepatic glucose production** **(mg.kg^-1^.min^-1^)**	1.8 ± 0.2	1.2 ± 0.3	1.8 ± 0.1

**Table 4:** Fasting plasma concentrations of lipid metabolites, hormones and inflammatory markers in diabetic pigs fed saturated fat/cholesterol or unsaturated fat or starch enriched diets

	Saturated fat plus cholesterol diet	Unsaturated fat diet	Starch diet
**NEFA (mmol/L)**	1.09 ± 0.17^A^	0.80 ± 0.14^AB^	0.58 ± 0.14^B^
**Triglycerides (mmol/L)**	0.58 ± 0.17	0.51 ± 0.05	0.43 ± 0.11
**Total cholesterol (mmol/L)**	9.9 ± 1.2^A^	2.0 ± 0.1 ^B^	1.7 ± 0.2 ^B^
**HDL cholesterol (mmol/L)**	2.1 ± 0.2^A^	0.4 ± 0.0 ^B^	0.5 ± 0.0 ^B^
**LDL cholesterol (mmol/L)**	3.3 ± 0.3^A^	1.3 ± 0.1 ^B^	1.1 ± 0.1 ^B^
**VLDL cholesterol (mmol/L)**	4.5 ± 0.8^A^	0.2 ± 0.1 ^B^	0.1 ± 0.0 ^B^
**Ratio HDL/LDL cholesterol**	0.64 ± 0.08	0.35 ± 0.05	0.46 ± 0.03
**Ratio HDL/total cholesterol**	0.21 ± 0.01^A^	0.23 ± 0.02^A^	0.29 ± 0.01 ^B^
**Glucagon (pmol/L)**	131 ± 25	114 ± 35	83 ± 10
**Cortisol (nmol/L)**	334 ± 44	284 ± 69	262 ± 50
**Haptoglobin (pg/mL)**	251 ± 132	394 ± 100	236 ± 52
**IL-6 (pg/mL)**	2224 ± 1102	818 ± 664	459 ± 270
**TNF-α (pg/mL)**	610 ± 289	394 ± 248	384 ± 135

^A, B ^, data with different superscripts in the same row differ at p < 0.05.

### Inflammation

**Figure 4:**
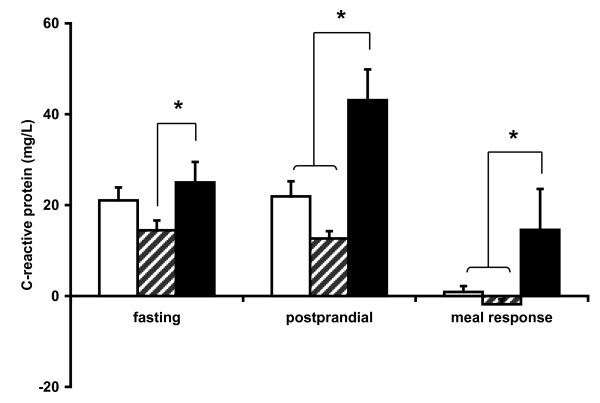
**Overnight fasting and postprandial (3 h) plasma C-reactive protein (CRP) concentrations and the meal response over fasting (Δ) are depicted for diabetic pigs which were fed diets supplemented with starch (open bars), unsaturated fats (striped bars) or saturated fats with cholesterol (black bars)**. Means ± SEM. *p < 0.05.

The fasting plasma concentrations of cortisol, haptoglobin, IL-6 and TNF-a were not statistically different between the diet groups (Table 4). By contrast, both fasting and postprandial plasma C-reactive protein (CRP) concentrations and meal-induced CRP responses (Δ) were higher (p < 0.05) in SFC pigs compared to UF pigs but not always higher compared to S pigs, i.e. fasting plasma CRP concentration was similar in SFC and S pigs (Figure 4).

### Atherosclerosis and blood pressure

**Figure 5:**
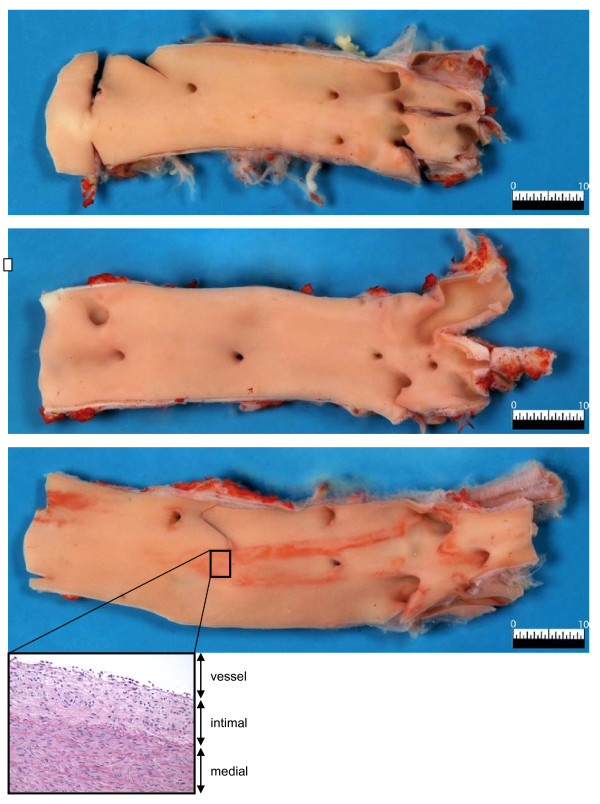
**Images of typical examples of abdominal aorta Sudan IV fat staining of diabetic pigs, which were fed diets supplemented with starch (top picture), unsaturated fats (middle), or saturated fats with cholesterol (bottom)**. Red intra-luminal staining indicates presence of fatty streaks. Insert in bottom picture shows histological detail (hematoxylin-eosin staining) of fatty streak (4 × magnification). The intimal area of this part of the vessel wall consists of multiple cellular layers with empty little spaces where fat was deposited.

**Figure 6:**
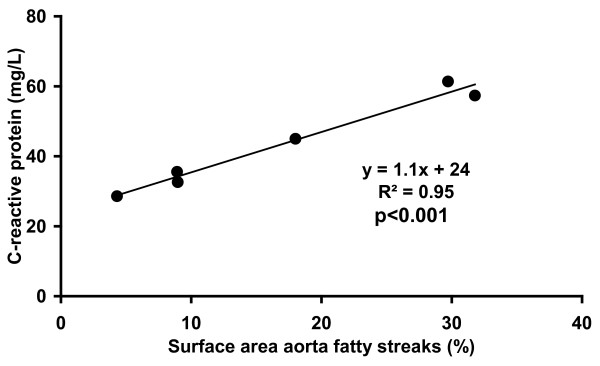
**Correlation between plasma C-reactive protein (CRP) concentrations and the surface area of abdominal aorta fatty streaks (%) in diabetic pigs which were fed a diet supplemented with saturated fats and cholesterol**. (R^2 ^= 0.95, p < 0.001).

The surface area of aorta fatty streaks was higher (p < 0.001) in SFC pigs compared to UF and S pigs (Table 2). Visualization of the fatty streaks in the abdominal aortas of the three groups of pigs is presented in Figure 5. In SFC pigs, a positive correlation between surface area of aorta fatty streaks and postprandial plasma CRP concentration was observed (R^2 ^= 0.95, p < 0.001) (Figure 6). Mean arterial, systolic and diastolic blood pressures were not statistically different between the diet groups (Table 5).

**Table 5:** Blood pressure and heart rate in diabetic pigs fed saturated fat/cholesterol or unsaturated fat or starch enriched diets

	Saturated fat plus cholesterol diet	Unsaturated fat diet	Starch diet
**Mean arterial pressure** **(mmHg)**	113 ± 14	96 ± 5	96 ± 4
**Systolic pressure (mmHg)**	139 ± 18	117 ± 5	116 ± 5
**Diastolic pressure (mmHg)**	97 ± 13	78 ± 5	78 ± 4
**Heart rate (beats per minute)**	109 ± 10	92 ± 21	109 ± 22

## Discussion

### Main findings

Pigs on dietary SFC showed a more atherogenic plasma profile with higher plasma NEFA concentrations, higher liver weights and liver triglyceride concentrations, and increased aorta fatty streak area compared to pigs on dietary UF and S. The postprandial glucose, but not insulin and triglyceride responses were intermediate in SFC pigs, lowest in UF pigs and highest in S pigs, whereas hepatic and whole body insulin sensitivities for glucose metabolism were not significantly affected by the diets. This indicates that the postprandial glucose intolerance of S pigs compared to SFC and UF pigs is caused by an increased systemic influx of dietary glucose after starch digestion and not by insulin resistance. The mechanisms behind the relative postprandial glucose intolerance in SFC pigs compared to UF pigs are unknown. However, several non-significant factors may contribute to the difference in postprandial glucose tolerance. Fasting and insulin-inhibited hepatic glucose production during the clamp were elevated by 16% (p = NS) and 50% (p = NS) and early (30 min) postprandial insulin secretion was 2-fold reduced (p = NS) in SFC pigs compared to UF pigs. For the latter, it has been suggested that the degree of saturation of fatty acids may have a negative influence on insulin release by beta-cells in vitro [34]. Taken together, these factors may add-up and act in concert to both impair non-insulin-dependent and insulin-dependent glucose tolerance in SFC pigs compared to UF pigs.

With respect to body composition, SFC pigs showed increased muscle, liver and aorta fat deposits and a reduced amount of retroperitoneal fat. The latter non-ectopic depot of adipose tissue was largest in UF pigs, indicating that body composition is most favourably affected by the UF diet compared to the SFC diet in this non-obese diabetic pig model. The UF diet is mainly stored in adipose tissue and to a lesser extent in organs and the vascular system. By contrast, the S pigs showed the lowest muscle and liver fat deposits and a low amount of retroperitoneal fat. This indicates that energy storage in the form of fat is low from dietary S, both in adipose and non-adipose tissue. The baseline markers of systemic inflammation tended to be higher in SFC pigs, but only baseline and postprandial CRP concentrations reached levels of statistical significance. A strong correlation was observed between postprandial plasma CRP and aorta fatty streak area (R^2 ^= 0.95, p < 0.001), suggesting that postprandial CRP is a biomarker for atherosclerosis in this diabetic pig model. This is one of the few pieces of evidence that the inflammatory marker CRP is secondary to the atheromatous process itself.

Taken together, these main findings indicate that dietary saturated fat/cholesterol induces CRP associated early atherosclerosis and ectopic fat deposition whereas eucaloric unsaturated fat or starch do not induce these abnormalities and unsaturated fat shows beneficial effects on postprandial glycaemia in diabetic pigs.

### Insulin resistance

After a 10-week intervention study, dietary composition had no significant effect on insulin sensitivity in diabetic pigs. This is in line with a 2-week intervention study in type 2 diabetic patients [35]. In non-diabetic rodents however, it is widely accepted that long-term feeding of dietary saturated fats induces insulin resistance whereas unsaturated fats increase insulin sensitivity [6]. This discrepancy may be caused by the fact that insulin resistance is already a prominent feature of diabetic humans [27] and pigs [19] and any modest dietary effect on insulin sensitivity does not come to expression in insulin resistant individuals. For example, both liver [6] and skeletal muscle [36] triglyceride concentrations have been shown to affect insulin sensitivity but in the present study, the diet-induced 2-fold variation in liver and skeletal muscle triglyceride concentrations is insufficient to further modulate insulin resistance in diabetic pigs.

### Inflammation

Fasting plasma concentrations of the acute phase protein CRP were higher in diabetic pigs which were fed the SFC diet compared to the UF diet but not to the S diet. This underlines the anti-inflammatory effect of dietary UF [37,38]. The pro-inflammatory effect of a diet rich in saturated fats and cholesterol has been shown before in non-diabetic humans [8,10] and in non-diabetic pigs [39,40]. In our study, postprandial plasma CRP, but not haptoglobin, IL-6 or TNF-α, positively correlated with the severity of beginning atherosclerosis. It seems therefore that postprandial CRP concentrations, which were increased 2-fold compared to fasting concentrations, react strongly to vascular damage and/or atherosclerosis [41]. Indeed, CRP has been proposed as a putative clinical biomarker for atherosclerosis [42,43] serving a possible protective role in the atherogenic process [44]. In diabetic pigs which were fed a diet rich in UF, no increase in postprandial CRP was observed. This corresponds with the absence of atherosclerosis in these diabetic pigs. The beneficial mechanism of action of UF, but not SFC, may be related to the affinity to bind to the nuclear peroxisome proliferator-activated receptor (PPAR), the same pathway which is activated by the anti-diabetic drug thiazolidinedione (TZD) [27,45-47]. Therefore, dietary UF can be regarded as a therapeutic agent to prevent some of the abnormalities of type 2 diabetes.

### Diabetic pig model

Fasting plasma insulin (~25 pmol/L), glucose (~13 mmol/L) and triglyceride (~0.5 mmol/L) concentrations and a positive energy balance (body weight gain of ~1.5 kg/week) without the occurrence of ketosis indicated a mild form of diabetes in the pigs. In a previous paper we argued that the present insulin resistant pig model resembled type 2 diabetes [19]. In addition to these observations, we now showed that, after streptozotocin treatment, the residual beta-cells of the pancreas were able to increase insulin secretion ~5-7 fold upon a meal-challenge. This indicates that a substantial endogenous insulin secretion capacity is still present in this diabetic pig model.

## Conclusion

When comparing dietary SFC, UF and S, dietary SFC induces CRP-associated early atherosclerosis and ectopic fat deposition whereas isoenergetic UF shows beneficial effects on postprandial glycaemia, inflammation and body composition in diabetic pigs.

## Abbreviations

AUC: area under the curve; CRP: C-reactive protein; ME: metabolizable energy; NEFA: non-esterified fatty acids; PPAR: peroxisome proliferator-activated receptor; PUFA: polyunsaturated fatty acids; S: starch; SFC: saturated fat with cholesterol; STZ: streptozotocin; TZD: thiazolidinedione; UF: unsaturated fat.

## Competing interests

The authors declare that they have no competing interests.

## Authors' contributions

SJK was the principal investigator, involved in designing the study and writing the manuscript. RD coordinated the study and performed statistical analyses. MTA, HS and MJS were involved in developing the clamp technique in pigs and performing the stable isotope measurements and writing parts of the manuscript. HMMB, MH and WJG were involved in the cardiovascular measurements and writing parts of the manuscript. All authors participated in writing the final version of the manuscript.
